# Does an active play standard change childcare physical activity and healthy eating policies? A natural policy experiment

**DOI:** 10.1186/s12889-022-13079-y

**Published:** 2022-04-08

**Authors:** Claire N. Tugault-Lafleur, Patti-Jean Naylor, Valerie Carson, Guy Faulkner, Erica Y. Lau, Luke Wolfenden, Louise C. Mâsse

**Affiliations:** 1 School of Nutrition Sciences, Faculty of Health Sciences, The University of Ottawa, 25 University Private, Ottawa, ON K1N 6N5 Canada; 2 School of Exercise Science, Physical and Health Education, Faculty of Education, University of Victoria, Victoria, BC V8W 3P1 Canada; 3 Faculty of Kinesiology, Sport, and Recreation, Van Vliet Complex, University of Alberta, 1-151 University Hall, Edmonton, AB T6G 2H9 Canada; 4 School of Kinesiology, University of British Columbia, 210-6081 University Blvd, Vancouver, BC V6T 1Z1 Canada; 5 Department of Emergency Medicine, Faculty of Medicine, University of British Columbia, 828West 10th Avenue, Vancouver, BC V5Z 1M9 Canada; 6 School of Medicine and Public Health, University of Newcastle, University Drive, Callaghan, NSW 2308 Australia; 7 School of Population and Public Health, Faculty of Medicine, University of British Columbia, Vancouver, BC V6T 1Z3 Canada; 8 School of Population and Public Health, BC Children’s Hospital Research Institute, F508-4490Oak Street, Vancouver, BC V6H 3V4 Canada

**Keywords:** Physical activity, Healthy eating, Obesity prevention, Childcare, Early years, Policy change

## Abstract

**Background:**

In 2017, the provincial government of British Columbia (BC) implemented a mandatory policy outlining Active Play Standards (AP Standards) to increase physical activity (PA) levels, sedentary and motor skills among children attending licensed childcare centers. Concurrently, a capacity-building initiative was launched to help implement policies and practices supporting both PA and healthy eating (HE) in the early years. This study evaluated differences in center-level PA and HE policies and practices before and after the enforcement of the new provincial AP Standards.

**Methods:**

Using a repeat cross-sectional design, surveys were distributed to managers and staff of licensed childcare facilities serving children aged 2–5 years before (2016–2017 or ‘time 1’) and after (2018–2019 or ‘time 2’) implementation of the AP Standards across BC. The total sample included 1,459 respondents (910 and 549 respondents at time 1 and time 2, respectively). Hierarchical mixed effects models were used to examine differences in 9 and 7 PA/sedentary policies and practices, respectively, as well as 11 HE policies between time 1 and time 2. Models controlled for childcare size and area-level population size, education, and income.

**Results:**

Compared to centers surveyed at time 1, centers at time 2 were more likely to report written policies related to: fundamental movement skills, total amount of Active Play (AP) time, staff-led AP, unfacilitated play/free play, total amount of outdoor AP time, limiting screen time, breaking up prolonged sitting, staff role modeling of PA, and training staff about PA (*P* < 0.01 for all 9 policies examined). Compared to time 1, centers at time 2 reported more frequent practices related to ensuring children engaged in at least 120 min of AP, 60 min of outdoor AP daily, and limiting screen time (*P* < 0.01 for 3 out of 7 practices examined). Despite no additional policy intervention related to HE, centers were more likely to report having written policies related to: HE education for children, encouraging new foods, having family-style meals, offering only milk or water, limiting the amount of juice served, staff role modeling of HE, limiting the types of foods at parties/celebrations and foods brought from home (*P* < 0.05 for 9 out of 11 HE policies).

**Conclusion:**

Approximately a year after the implementation of a governmental policy targeting PA supported by a capacity-building initiative, childcare centers reported positive changes in all 9 PA/sedentary policies examined, all 3 out of 7 PA/sedentary practices and 9 out of 11 HE policies evaluated at the center-level.

## Background

Ensuring sufficient and quality physical activity (PA) and nutrition in the early years is key to preventing excessive weight gain as well supporting healthy children’s physiological, psychosocial, and educational outcomes [1–3]. Providing opportunities for PA, motor development and healthy eating (HE) in early childhood can, over the lifespan, influence the maintenance of a healthy active lifestyle and play a role in disease prevention. Yet in Canada, evidence suggests room for improving preschoolers’ PA and sedentary behaviours [4, 5] as well as overall dietary quality [6–8].

Childcare settings are an amenable and promising environment to facilitate the delivery of obesity prevention programs as they provide access to large numbers of children during a critical period of their growth and maturation [9, 10]. In Canada, more than half (54%) of parents with children under age 5 are using childcare, and of these children, 70% are in full-time (at least 30 h per week) childcare [11], leaving limited time outside of childcare for PA participation. The newly released Canadian 24-Hour Movement Guidelines for the Early Years (0–4 year) states young children should engage in a minimum of 180 min of any-intensity PA per day (of which at least 60 min should be energetic play or moderate to vigorous physical activity) and screen time limited to no more than 60 min/day for children 2–4 years [12]. Yet studies examining PA of children within these settings suggest that the PA during childcare hours is insufficient (i.e., preschoolers are getting between 12 and 14 min of moderate-to-vigorous PA per day) with little opportunities to develop fundamental movement skills (FMS) [13–15]. Although research examining the quality of foods consumed at daycares in Canada is scant, studies documenting the quality of food offerings [7, 16] equally suggests room for improvement.

Empirical evidence suggests that changes in PA policies can lead to significant improvements in childcare environments and providers’ practices with respect to PA [17–21] while the evidence on the effectiveness of nutrition policies on children’s dietary intakes is more equivocal [20]. Evaluations of such programs can be challenging due to their large scale, resources constraints, and challenges associated with measuring change over time, making it difficult to identify strong, generalizable evidence to improve policy design and implementation [22]. Consequently, researchers and practitioners have called on the importance of evaluating natural policy experiments aimed at changing nutrition and PA behaviours [23, 24].

In British Columbia (B.C.) (Canada), regulations guiding PA and nutrition practices for licensed childcare settings have been in place since 2007 and are regularly updated through the Community Care and Assisted Living Act: Child Care Licensing Regulations [25]. In July 2016, the provincial government of BC announced a new policy outlining standards for active play (AP) and sedentary time policies and practices: the Director of Licensing Standard of Practice for Active Play (or simply, the Active Play Standards (AP Standards) [26]. An educational/information dissemination year followed the announcement. The AP Standards were then fully enforced in July 2017 and included the following standards of practice for active play: 1) a minimum of 60 min per day of outdoor AP; 2) the incorporation of FMS and injury prevention in all AP activities; 3) limits on screen time and prolonged sitting; 4) role modeling of staff of AP and screen time; and 5) the development of written AP and screen time policies [26]. Under this new standard, facilities are audited approximately every 18 months and licensing officers log any contraventions and identify goals and a timeframe within which the childcare provider is expected to bring their center into compliance [26]. Concomitant with the release of the AP Standards, the provincial roll-out of a capacity-building initiative for early years providers, entitled Appetite to Play (ATP) was launched in the summer of 2017 [27]. The purpose of ATP was to ensure early years providers would have sufficient capacity to implement evidence-based policies, practices, and environments to support PA and HE, and support compliance with the AP Standards [27]. ATP was developed for dissemination at scale and based on existing evidence and resources customized for stakeholder needs instead of a researcher-driven process mobilizing a specific intervention from pilot testing to efficacy, effectiveness, and then scale-up [28]. Previous research has highlighted the widespread roll-out of ATP, which covered half of BC municipalities and was estimated to reach ~ 2,700 early year providers over 18 months [27]. While the AP Standards focused on PA in the early years, the inclusion of HE resources for early year providers was unique to ATP and there was no mandatory provincial HE standard released as with the AP Standards. The ATP initiative included: both in-person and online training workshops for managers and staff with content related to PA and HE in the early years (including physical and food literacy), a web-based toolkit that was updated weekly and provided interactive tools like center audits, weekly PA and meal planners, recommended practices, AP and HE related ideas and tips, a community of practice and a communications and marketing initiative.

The objectives of this study were to: 1) describe the level of implementation of the AP Standards in a sample of childcare centers across BC; and, 2) examine differences in the prevalence of centers with written policies and frequency of reported practices related to PA, sedentary and HE behaviours in 2018–19 compared to 2016–17. Following the implementation of the AP Standards and scale-up of ATP training, we hypothesized that a greater proportion of childcare centers would have written PA and report more frequent practices targeted by the AP Standards (more total AP, outdoor AP, FMS activities, modeling PA by staff, limits on screen time, and breaking up prolonged sitting). Since the implementation of the AP Standards did not focus on nutrition or HE, we did not have an a priori hypothesis regarding changes in centers’ HE policies over time.

## Methods

All methods were performed in accordance with the relevant guidelines and regulations and the study was approved by the University of Victoria and University of British Columbia Harmonized Research Ethics Review Board (BC16-128 and H18-01,434). Respondents gave their implicit and informed consent by answering the Early Years survey.

### Participants and recruitment

The study sample for this repeat cross-sectional study included managers and staff of licensed childcare centers serving children 2–5 years of age across British Columbia (BC), Canada. Respondents were eligible to participate if their center cared for children age 2 ½ -5 years old, was licensed for group childcare, preschool (offering full days), and/or multi-age childcare and if the respondent was a manager or a staff caring for children age 2 ½—5 years old, respectively.

The first wave of online data collection started from October 2016 and ended in August 2017. At baseline, the only standards in place included the size of the play space and providing opportunities for gross motor movements [25]. The AP Standards, officially released in July 2016, were not mandatory until a year later in the summer of 2017. The second wave of survey recruitment took place from October 2018 to September 2019, approximately one year after the enforcement of the AP Standards and the launch of ATP. Recruitment strategies included initial emails to managers from licensing officers and direct mail, email, and phone calls from the research team using publicly available center contact information as well as invitations distributed through childcare resource and referral agencies and early childhood educator newsletters. Childcare managers were asked to forward the staff invitation and survey links to their staff. Childcare center managers who did not respond to initial and follow-up email invitations were sent a paper copy of both the manager and two staff surveys with pre-paid postage-stamped envelopes for return. Managers who had not yet responded also received phone calls from our research team and were offered the choice of electronic or paper survey for themselves and their staff.

A total of 1,459 participants (*n* = 910 in 2016–17 (‘time 1’) and *n* = 549 in 2018–19 (‘time 2’)) were included in our sample. These respondents worked at 592 and 378 centers at time 1 and time 2, respectively. Based on a sampling frame of approximately 1,500 registered licensed childcare centers in BC, our response rate was approximately 41% and 25% in 2016–17 and 2018–19, respectively. At time 1, 39% of the facilities (*n* = 232 out of 592 centers) had more than one respondent fill out the survey and included between 2 and 6 surveys for a given facility. At time 2, 29% of the facilities (*n* = 108 out of 378 centers) had more than one respondent fill out the survey and included between 2 and 5 surveys for a given facility.

### Measures

Respondents completed a self-report survey with items adapted from the validated EPAO-SR (Environment and Policy Evaluation and Observation Self-Report) instrument to include survey items about BC-specific childcare center characteristics [29]. Questions about daily practices related to a new active play standard, and demographic characteristics were developed by our research team based on feedback from a provincial Early Years HE and PA resource advisory committee, the research team, and a pilot group of early childhood educators (*n* = 7). In 2018–19, some survey items were also developed to ensure that the questions measured the policies and practices targeted by the AP Standards.

At time 1, separate versions of the Early Year Surveys were sent to managers and staff in each facility (manager and staff surveys). However, at time 2, a single version of the Early Year survey was sent out to a center and the respondent had a staff position, they were asked to answer additional questions related to PA/sedentary practices on the previous day. At both time points, respondents were asked to choose the position that best described their position/role at the facility. Respondents could select from 5 response options: 1) “Executive director/program manager”, 2) “staff that care for 2 ½ to 5-year-old children”, 3) “staff that care for children younger than 2 ½ or older than 5-year-old children”, 4) “Administrative assistant/office manager” or 5) “other”. Respondents were classified as “eligible managers” if they selected “Executive director/program manager” and/or “Administrative assistant/office manager”. Respondents were classified as “eligible staff” if they selected “staff that care for 2 ½ to 5-year-old children”. Respondents who selected “executive director/program manager”/ “Administrative assistant/office manager” as well as “staff that care for 1 ½ to 5-year-old children” were classified as “eligible manager and staff”. In order to aggregate responses within a center for our outcomes (as many centers had more than one manager or one staff fill out the survey), we also classified each survey as a “manager survey” or a “staff survey” based on the respondent’s position. Any survey from an “eligible manager” or “an eligible manager and staff” was classified as a “manager survey”; otherwise, the survey was classified as a “staff survey”.

#### Prevalence of center-level PA, sedentary and healthy eating policies

In 2018–19, both managers and staff were asked about the prevalence of policies related to physical activity and nutrition but in 2016–17, only managers were asked about the prevalence of policies. Managers (as well as staff in 2018–19) were asked: “Does your facility have a policy/guideline that includes a statement about…”: 1) staff provision of activities that addressed fundamental movement skills; 2) the total amount of AP time; 3) the amount of staff-led AP time; 4) the amount of unfacilitated play / free play; 5) the amount of outdoor AP time each day; 6) the amount of time children can play with or watch screens; 7) breaking up prolonged sitting time with activity; 8) staff role modeling of PA and screen time; 9) training staff about PA/developing physical literacy. Respondents could select from the following answer options: “no”, “yes, not written policy”, “Yes, written policy”, or “N/A”. Answers were recoded and dichotomized into: “yes, written policy” or “No written policy” (which included the response options of “yes not written” and “No”).

Related to HE policies, managers (as well as staff in 2018–19) were asked: “Does you facility policy include a statement about…”: 1) HE education for children; 2) HE training for staff; 3) encouragement of new foods; 4) family style meals (staff sit with children to enjoy food together); 5) offering drinking water or milk only; 6) type of milk served (1%, 2%, whole, flavoured); 7) amount of fruit juice served; 8) staff role modeling of eating behaviours; 9) encouraging inclusion of fruit/vegetable when snack/meal is served; 10) limiting types of food and drink brought to parties and celebrations; 11) types of beverages and/or food brought from home. Respondents could select from the following options: “No”, “Yes, not written policy”, or “Yes, written policy”. Responses were recoded into “Yes, written policy” or “No written policy” (which included the response options of “Yes, not written policy” and “No”).

When more than one respondent from a center responded to the survey (‘duplicates’), respondents’ answers were aggregated using the following protocol. For each policy item (e.g., “Does your facility has a policy/guideline that includes a statement about the total amount of AP?” yes written/no written policy), responses were averaged across respondents by year and position/role (manager or staff). If agreement among managers was greater than 66.7%, their policy score was rounded to either “0” (no written policy) or “1” (written policy). If agreement among managers was below 66%, then the survey response was recoded as missing. To determine the presence of a written policy within a center, we prioritized managers’ responses over staff. However, when manager responses were missing and policy responses were available from a staff, we used staff responses.

#### Frequency of PA and sedentary practices

At both time points, managers and staff were asked to indicate how frequently children in their program: 1) took part in daily activities that develop fundamental movement skills; 2) engaged in at least 120 min of AP and PA per day; 3) engaged in at least 60 min of outdoor active play per day; 4) spent 30 min or less on screens per day; 5) see staff being active; 6) did not sit for prolonged periods (e.g., board games, crafts, etc.); and 7) learn about why PA is good for them. Response options for each of the practices included “Daily”, “Most days (3–4)”, “Some days (1–2)”, “infrequently”, and “rarely/never”. Responses were coded on a 1–5-point Likert scale, with a higher score indicating a more frequent practice.

When more than one respondent from a center responded to the survey (‘duplicates’), these duplicates were processed as follows. For each practice item, responses were averaged by year and by position/role (managers or staff). Our research team had previously explored consistencies and discrepancies in self-reported policies and practices between managers and staff [29]. We had found similar prevalence estimates between manager- and staff-reported PA practices that are typically scheduled [29]. However, due to the nature of their administrative role and limited direct experience in the classroom, we prioritized practice responses from staff over those from managers. When staff responses for practices were missing and manager-reported practices were available, we supplemented our missing data using response from manager surveys.

#### Other respondent-, center- and area-level characteristics

In addition to their role/position in the facility, care providers were asked about their age, gender, and number of years working at the facility. At the center level, we inquired centers about the current number of number of children enrolled, their food procurement patterns (i.e., whether the food was all brought from home, provided by the center or mixed in terms of source meaning some foods were brought from home and some was provided by the center) and about the level of food preparation done at the facility, with response options ranging from “Hot and cold snacks prepared on site on all or most days”, “minimal food preparation (e.g., slicing fruit, cheese), “pre-packaged single portion foods”, “all food supplied by parents” or “Other”. At time 2 (but not time 1), we also asked centers to indicate what food provisioning system was used for AM snack, lunch, and PM snack (i.e., respondents were asked whether food was provided by the center or brought from home). Population size, percentage of the population with some post-secondary education and median household income were obtained from the Statistics Canada 2016 Census and linked to childcare site data using postal codes.

### Statistical analyses

Descriptive statistics (means, standard deviations (SD) and proportions/percentages (%)) were used to describe the characteristics of respondents and childcare facilities, their AP Standards and ATP implementation status (e.g., whether they had a licensing officer review the center in the past year, whether they had attended ATP training), prevalence of centers with written policies at each time point, and mean scores for reported practices. Hierarchical mixed effects models were used to assess differences in the prevalence of centers reporting having written policies and frequency of reported practices between 2016–17 and 2018–19. Hierarchical mixed effects models were chosen to account for the unbalanced nature of the dataset: while most childcare centers provided only data at one time point (2016–17 or 2018–19), 146 centers provided data at both time points. Finally, sensitivity analyses were conducted to explore whether the prevalence of policies changed when taking only responses from managers (as opposed to supplementing missing manager responses with staff surveys). The same sensitivity analyses were conducted for practices using only responses from staff (as opposed to supplementing missing staff responses with manager surveys).

All analyses were conducted in STATA version 16 [30]. Analyses were conducted using the XTMIXED (linear outcomes) or XTMELOGIT (for binary outcomes) commands. This procedure is more powerful than examining only childcare centers that provided data in both 2016–2017 and 2018–2019 and assumptions for missing data are weaker (missing data at random vs. missing completely at random). Adjusted models controlled for number of children enrolled in the center and area level population size, proportion of the population with some post-secondary education, and total household income. A *p*-value of less than 0.01 was considered statistically significant.

## Results

### Respondents and childcare characteristics

Participant and childcare center characteristics (*n* = 1,459 respondents from 970 centers) are shown in **Table ****1**. The proportion of managers and staff was similar across survey cycles, with about 60% of surveys being filled by respondents occupying a managerial position and 40% occupying a staff position within the centers. The distribution of respondents’ age and gender profile was similar between survey periods. At time 1 and 2, 77% and 72% of respondents, respectively, reported having worked at the center for 5 years or more.

**Table 1:** Respondents and center-level characteristics before and after implementation of the Active Play Standard in British Columbia

	**Survey cycle**	
	**2016–2017**	**2018–2019**
*Respondent characteristics*		
Respondents, n (%)	910 (62.4)	549 (37.4)
Sex, n (%)		
Female	884 (97.1)	470 (89.9)
Male	19 (2.1)	16 (3.1)
Prefer not to disclose	7 (0.8)	37 (7.1)
Position, n (%)^a^		
Manager/director/administrator	549 (60.3)	322 (58.7)
Childcare staff	361 (39.7)	227 (41.4)
Age group, n (%)		
< 30 years	122 (13.4)	78 (14.2)
30–39 years	237 (26.0)	162 (29.5)
40–49 years	266 (29.2)	134 (24.4)
50–59 years	202 (22.2)	95 (17.3)
> 60 years	83 (9.1)	80 (14.6)
Number of years working at center, n (%)		
1–5 years	206 (22.6)	156 (28.4)
5 years or more	704 (77.4)	393 (71.6)
*Center-level characteristics*		
Centers, n (%)^b^	592 (61)	378 (39)
Center enrollment, mean (SD)	37 (28)	38 (37)
Food provisioning, n (%)		
All food brought from home	183 (36.7)	131 (35.6)
Center provides all foods	73 (14.6)	65 (17.7)
Mixed source	243 (48.7)	172 (46.7)
Food processing/preparation		
Hot and cold snacks and/or meals prepared onsite	177 (35.5)	136 (37.1)
Minimal food preparation (e.g., cutting fruit)	144 (28.9)	97 (26.4)
Pre-packaged (single portion) foods	7 (1.4)	3 (0.8)
All foods supplied by parents	157 (31.5)	114 (31.1)
Other	14 (2.8)	17 (4.6)
Food provisioning at lunch^c^, n (%)		
Provided by center, prepared on site	-	69 (19.2)
Provided by center, prepared off site	-	15 (4.2)
Must be brought from home	-	254 (70.6)
Not served	-	22 (6.1)
Food provisioning at morning snack^c^, n (%)		
Provided by center, prepared on site	-	199 (55.0)
Provided by center, prepared off site	-	5 (1.4)
Must be brought from home	-	145 (40.1)
Not served	-	13 (3.6)
Food provisioning at afternoon snack^c^, n (%)		
Provided by center, prepared on site	-	190 (51.9)
Provided by center, prepared off site	-	10 (2.7)
Must be brought from home	-	147 (40.2)
Not served	-	19 (5.2)
*Area-level characteristics*		
Percent of population with some postsecondary education, % (SD)	62 (13)	63 (13)
Medium household income (CAN$), mean (SD)	87,542 (23,056)	88,201 (22,817)

^a^Respondents were asked to choose the position that best described their role at the facility and included 5 response options: 1) “Executive director/program manager”, 2) “staff that care for 2 ½ to 5-year-old children”, 3) “staff that care for children younger than 2 ½ or older than 5-year-old children”, 4) “Administrative assistant/office manager” or 5) “other”. Respondents were classified as “eligible managers” if they selected options 1 (“Executive director/program manager”) and/or option 4 (“Administrative assistant/office manager”). Respondents were classified as “eligible staff” if they selected option 2 (“staff that care for 2 ½ to 5-year-old children”). Respondents who selected both option 1 (“executive director/program manager” and option 2 (“staff that care for 1 ½ to 5-year-old children”) were classified as “eligible manager and staff”

^b^A total of 592 and 398 facilities answered the surveys in 2016–17 and 2018–19, respectively but within this sample, 146 facilities returned and completed the survey at both time points

^c^Only assessed at time 2 (2018–19) but not at time 1 (2016–17)

Childcare centers surveyed at time 1 and 2 had on average 37 and 38 children enrolled at their centers, respectively. In both survey years, just under half of the centers (49% and 47%) reported a mixed childcare food provisioning system (e.g., the center provided some foods, and some foods were brought in from home). Only 15% and 17% of the centers surveyed at time 1 and time 2, respectively, provided all the food for children in the center. A total of 37% and 36% of centers asked parents to bring in all foods for the day at the center at both time points. The extent to which centers prepared food for children was similar between survey years, with ~ 36% and 37% of centers preparing hot and cold meals and/or snacks on site, 29% and 26% reporting minimal food preparation on site (e.g., slicing applies, cutting cheese) at time 1 and time 2, respectively. At time 2 (when centers were asked about food procurement patterns for morning snack, lunch and afternoon snack), most centers (77%) reporting having children bring in their own packed lunch from home. Having a center provide morning or afternoon snacks was more common than providing lunch, with just over half of centers providing a snack (morning and/or afternoon) to children. Facilities were in communities with similar socioeconomic profiles (62% and 63% of households with some post-secondary education and a median household income of CAN $87 k) in both survey years.

### Implementation of the AP Standards

At time 2, most respondents (96%) reported that their facility had been reviewed by a licensing officer within the past year and 89% of respondents confirmed that the licensing officer had reviewed whether their facility met the AP Standards. A total of ~ 18% of respondents reported that the licensing officer had recommended to make some changes to meet the new AP Standards. Just under a quarter of respondents (22%) surveyed in 2018–19 also reported attending the ATP training workshops. Among those who completed the ATP training (*n* = 119 participants), 83% of participants completed the training in person vs. 17% of participants who reported taking part only in online training.

### Differences in policies and practices before and after implementation of the AP Standards

The prevalence of PA, sedentary and HE policies and frequency of PA and sedentary practices at time 1 and time 2 are shown in **Table ****2****.** Before the implementation of the AP Standards, the prevalence of written center-level policies on PA and sedentary behaviours ranged from 8% (policies related to staff role modeling of PA and screen time) to 43% (policies related to amount of daily outdoor active play). After the implementation of the AP Standards, the prevalence of PA/sedentary policies ranged from 17% (policies related to training staff about PA and physical literacy) to 77% (policies related to daily outdoor active play time). At time 1, average scores for PA/sedentary practices ranged from 3.0 (limiting screen time) to 4.6 (engaging in at least 60 min of outdoor AP daily) (out of a possible scale of 1 to 5 points). At time 2, practices scores ranged from 3.6 (spending less than 30 min on screens daily) to 4.9 (engaging in at least 60 min of outdoor AP daily.

**Table 2:** Prevalence of center-level policies and reported practices before and after implementation of the Active Play Standards in British Columbia, Canada (*n* = 592 centers in 2016–17 and *n* = 378 centers in 2018–19)

	2016–17 *n* = 592 centers		2018–19 *N* = 378 centers	
**Physical activity/sedentary policies**^**a**^	**n**	**%**	**n**	**%**
Provision of activities that address fundamental movement skills	501	15.0	361	58.2
Total amount of active play time	496	39.1	360	74.2
Amount of staff-led active play	500	18.2	364	30.0
Amount of un-facilitated play / free play	499	33.1	35	47.9
Amount of daily outdoor active play	497	42.5	363	77.4
Amount of screen time	499	25.7	359	70.8
Breaking up prolonged sitting	499	17.2	363	26.2
Staff role modeling of physical activity and screen time	499	7.9	358	36.3
Training staff about physical activity and/or physical literacy	501	9.6	363	17.1
**Healthy eating policies**^**a**^				
Healthy eating education for children	498	51.8	360	68.9
Healthy eating training for staff	499	15.0	366	18.6
Encouragement of new foods	498	20.3	358	28.5
Family style meals (staff sit with children)	498	29.1	358	35.8
Offering drinking water or milk only	497	50.1	360	57.2
Types of milk served	501	13.6	365	15.3
Amount of fruit juice served	499	11.8	359	18.4
Staff role modeling of eating behaviours	500	19.6	357	29.7
Encouraging inclusion of fruit/vegetable	496	46.4	361	56.5
Limiting types of foods at parties/celebrations	498	25.5	353	36.5
Types of foods and beverages brought from home	497	23.4	360	53.1
**Physical activity/sedentary practices**^**b**^		**Mean (SD)**		**Mean (SD)**
Take part daily in activities to develop fundamental movement skills	535	4.6 (0.8)	349	4.8 (0.6)
Engage in ≥ 120 min of active play daily	534	4.5 (0.9)	349	4.7 (0.6)
See staff being active	535	4.5 (0.9)	346	4.6 (0.7)
Spend 30 min or less on screens daily	523	3.0 (1.9)	346	3.6 (1.8)
Do not sit for prolonged periods	534	4.2 (1.3)	349	4.5 (1.1)
Engage in ≥ 60 min of outdoor active play daily	474	4.6 (0.8)	347	4.9 (0.5)
Learn why physical activity is good for them	533	4.1 (1.0)	347	4.4 (1.0)

^a^In 2018–19, both managers and staff were asked about the prevalence of policies related to physical activity and nutrition but in 2016–17, only managers were asked about the prevalence of policies. In centers where more than one manager or more than one staff answered the survey, their policy responses were aggregated by survey type (manager survey vs. staff survey) within a center for each time point (2016–17 or 2018–19). We prioritized manager(s)’s responses to determine the presence of written policies in a center. However, when manager responses were missing and policy responses were available from staff, we used staff responses

^b^All practice items were measured using a 5-point Likert scale ranging from “Rarely/Never” (= 1) to “Daily” (= 5). The higher the score, the more frequent the behaviour. We prioritized staff response(s) to determine the frequency of practices in a center. However, when staff responses were missing and practice responses were available from managers, we used manager responses

At time 1, the prevalence of HE policies ranged from 12% (policies related to the amount of fruit juice served to children) to 52% (policies related to providing HE education to children). At time 2, the prevalence of HE policies ranged from 15% (policies on the type of milk served) to 69% (policies related to HE nutrition education for children).

**Table ****3** displays the covariate-adjusted odds of centers reporting written PA/sedentary and HE policies and estimated differences in the frequency of PA/sedentary practices at time 2 compared to time 1. Centers reported differences in all 9 PA/sedentary policies and 3 out of the 7 PA/sedentary practices examined. The highest odds were for policies related to limiting screen time (AOR = 18.0), fundamental movement skills (Adjusted odds ratio (AOR) = 11.6), staff role modeling of AP (AOR = 6.9) and ensuring a total amount of AP time (AOR = 5.7). Centers at time 2 also reported more frequent practices related to providing at least 120 min of AP daily (adjusted β = 0.2 (95% CI: 0.1, 0.3)), 60 min of outdoor AP daily (adjusted β = 0.2 (95% CI: 0.1, 0.3)), and limiting screen time to no more than 30 min daily (adjusted β = 0.6 (95% CI: 0.3, 0.8)).

**Table 3:** Changes in center-level physical activity, sedentary and healthy eating policies and practices before and after implementation of the Active Play Standards in British Columbia, Canada (*n* = 970 centers)^a^

**Physical activity/sedentary policies**	**n**	**AOR (95% CI)**
Provision of activities that address fundamental movement skills	784	**11.6** (6.2, 21.5)**
Total amount of active play time	783	**5.7** (3.6, 9.1)**
Amount of staff-led active play time	795	**2.4** (1.5, 3.9)**
Amount of un-facilitated play / free play	785	**2.0** (1.4, 3.0)**
Amount of daily outdoor active play time	789	**5.5** (3.5, 8.6)**
Amount of screen time	783	**18.0** (7.5, 43.0)**
Breaking up prolonged sitting	793	**1.7** (1.2, 2.6)**
Staff role modeling of PA and screen time	788	**6.9** (3.8, 12.6)**
Training staff about PA and physical literacy	799	**2.5** (1.3, 4.8)**
**Healthy eating policies**	**n**	**AOR (95% CI)**
Healthy eating education for children	784	**2.3** (1.5, 3.5)**
Healthy eating training for staff	794	1.5 (0.9, 2.4)
Encouragement of new foods	787	**1.7** (1.1, 2.6)*
Family style meals (staff sit with children)	784	**1.7** (1.1, 2.7)*
Offering drinking water or milk only	787	**1.4** (1.0, 2.0)*
Types of milk served	797	1.2 (0.8, 1.9)
Amount of fruit juice served	791	**1.9** (1.2, 3.0)**
Staff role modeling of eating behaviours	787	**2.0** (1.3, 3.1)**
Encouraging fruit/vegetable	785	**1.6** (1.1, 2.2)*
Types of foods at parties/celebrations	780	**1.5** (1.0, 2.1)*
Types of foods brought from home	786	**5.0** (3.1, 8.0)**
**Physical activity practices**	**n**	**beta (95% CI)**
Take part daily in activities to develop fundamental movement skills	711	0.1 (-0.0, 0.2)
Engage in at least 120 min of active play and physical activity daily	711	**0.2** (0.1, 0.3)**
Engage in at least 60 min of outdoor active play daily	688	**0.2** (0.1, 0.3)**
Spend 30 min or less on screens daily	707	**0.6** (0.3, 0.8)**
See staff being active	707	0.0 (-0.1, 0.1)
Do not sit for prolonged periods	711	0.2 (-0.0, 0.3)
Learn why physical activity is good for them	709	0.1 (-0.0, 0.3)

*AOR* adjusted odds ratio, *CI* confidence interval

**Bolded odds ratios** are statistically significant at *p-value < 0.05 ** *p*-value < 0.01. Hierarchical mixed effect logistic and linear regression models were used to assess changes in policies (binary outcomes) and practices (continuous outcomes), respectively. Covariates included number of children enrolled and area-level community variables (population size, median income, and percent of individuals with some post-secondary education)

In terms of HE, centers were more likely to reporting having written policies for 9 of the 11 policies examined. Compared to time 1, centers at time 2 were more likely to report written policies related to: foods and beverages brought from home (AOR = 5.0), providing nutrition education to children (AOR = 2.3), staff role modeling of HE (AOR = 2.0), the amount of fruit juice served (AOR = 1.9), encouraging children to try new foods (AOR = 1.7), serving family-style meals (AOR = 1.7), encouraging fruit/vegetables at meal & snack times (AOR = 1.6), limiting the types of foods at parties/celebrations (AOR = 1.5), and offering water or milk only (AOR = 1.4).

## Discussion

Childcare centers are salient settings to institutionalize comprehensive health promoting policies, which possess the potential to lead to widespread changes in PA and HE [31]. In Canada, provinces and territories vary considerably in their obesity prevention regulations for young children [32]. Moreover, research shows that standards for childcare PA are largely lacking across Canada, varying across provinces [30] and within childcare centers [33]. To our knowledge, our study is the first Canadian study taking advantage of a natural policy experiment targeting the implementation of new AP Standards accompanied by a capacity-building intervention (ATP) delivered across the province. Our findings confirm that a year after the enforcement of a mandatory Active Play governmental policy supported by a capacity-building initiative, centers were more likely to report written policies related to PA/sedentary behaviours and centers reported smaller changes in some of the PA/sedentary behaviours evaluated.

Our study contributes to a limited body of evidence about the effectiveness of governmental policy change on childcare centers’ PA and HE policies and practices. Limited Canadian research has explored the institutionalization of PA guidelines in early year settings. A 2016–17 survey of childcare providers across Canadian provinces and territories found that less than half of centers surveyed (n = 514) reported having written policies related to PA and fewer (29%) had written policies related to screen time [33]. A recent PA policy RCT (the Childcare PLAY Policy study) conducted among childcare centers in Southwestern Ontario also suggested low levels of institutionalization of provincial-level policies within early year settings [34]. Findings from our study suggest that at baseline (in 2016–17), the prevalence of center-level written PA and sedentary policies was low, and similar to what has been reported in a national sample of childcare centers [33]. For example, centers surveyed at time 1 reported written PA policies ranging from 8% (for PA policies related to staff role modelling of PA) to 43% (for amount of daily outdoor active play). Monitoring was a key component of policy enactment in BC, with 97% of respondents reporting that their center had been reviewed by a licensing officer within the year following the enforcement of the AP Standards. Although our results suggest higher institutionalization of PA and sedentary provincial regulations into childcare centers following the enforcement of the AP Standards compared to the national average, it is also possible (and very likely) that that implementation of a new written policy was variable across centers.

There is limited evidence of the effectiveness of governmental policy change on childcare centers’ PA and HE policies and practices. A 2019 systematic review of natural policy experiments targeting childhood obesity prevention and control reported that of the 33 natural experiments, most (73%) took place in school settings [35]. In the U.S., a handful of studies have examined changes in childcare PA policies and practices following changes in state-wide regulations [17, 18, 36, 37]. In Louisiana, Kracht et al. [18] examined policy and practice changes in a cohort of centers over one year following state-level changes in childcare regulations (*n* = 9 ECE centers). While no statistically significant difference in total EPAO scores were found (*p* = 0.06), centers improved their EPAO sub-scores for providing more AP opportunities and improving staff behaviors. Another quasi-experimental study examining the effect of mandatory PA standards for ECE centers in South Carolina reported significant improvement in total EPAO scores over a one year period [17]. The latter study also used a comparison group (a group of ECE centers in North Carolina, a state that did not make any policy changes) and reported a state-by-time interaction term which approached significance (*p* = 0.06). In Australia, a longitudinal study assessing centers’ adoption of six PA and HE practices over a 7-year period (*n* = 358 centers) reported significant increases in the prevalence of services adopting all but one practice [38]. Combined with our findings, the existing literature suggests that state-level/province-wide mandatory PA policies do have some impact on policies at the facility level.

When examining the potential influence of capacity-building on policy implementation we found that a relatively small proportion of respondents (22%) reported attending the ATP workshops (attending either in-person or online), suggesting overall low uptake/adoption of ATP training in this sample of centers. Thus, it may be possible that the monitoring of the policy played a larger role in changing center-level PA/sedentary policies than implementation support strategies. However, a key component of the ATP intervention included a web-based toolkit (www.appetitetoplay.com), freely accessible to the public. Some of the respondents who reported not attending the ATP training could have accessed some of the web-based resources promoted by other early year providers who attended the training workshops. Moreover, the timing of the 2018–19 surveys also aligned with the release of the 24-Hour Movement Guidelines for the Early Years [12]. It is therefore possible that the release of these guidelines and passive access to web-based resources could have played a role in informing or influencing center-level policies surrounding PA.

From 2016–17 to 2018–19, childcare centers reported increased prevalence of written PA and sedentary policies, but the change over time was not consistent across all types of policies. The AP Standards provided explicit guidance regarding the minimum amount of time dedicated for AP, incorporating FMS activities as part of their daily indoor and outdoor routines and limiting screen time [26]. Not surprisingly, we found that the prevalence of center-level policies increased more substantially for the types of policies that related directly to these new AP Standards: limiting screen time, providing activities to develop FMS, daily outdoor play and staff role modelling of PA and screen time. Taken together, these findings highlight the importance of specificity within policy interventions.

Although BC childcare centers on average improved most of their PA and sedentary policies over a two-year period, changes in PA practices appeared to be very modest. An important limiting factor here could have been the high frequency of reported PA practices at baseline, leaving little room for improvement. It is also possible that factors at the childcare-level (e.g., organizational climate, PA capacity) and provider-level (e.g., level of training in physical literacy, self-efficacy) could influence the implementation of the AP Standards on PA practices. Previous implementation studies have highlighted the importance of additional resources to help teachers meet new PA regulations such as staff training and helping purchase portable equipment to promote AP [39]. Next steps in this research include examining what factors at the provider and center-level were associated with positive changes in policies and practices in the smaller cohort of centers who provided data both in 2016–17 and 2018–19.

The early years are an important time for developing eating skills and accepting a variety of healthy foods. Mealtimes should represent supportive environments for practicing skills and trying unfamiliar foods [40]. Our results suggest that over a two-year period, the prevalence of childcare centers with written HE policies increases substantially. These findings are encouraging, given evidence suggesting that most provinces/territories in Canada have relatively weak nutrition standards that lack specificity in the types of foods and beverages offered and served to children in care [32]. Whether changes in HE policies lead to meaningful changes in the childcare food environment (or to children’s eating behaviours) remains unknown. Canadian studies evaluating the nutritional quality of foods served in childcare settings, mealtime behaviours or caregivers and provision of nutrition education are scarce. Available international evidence suggest implementation strategies can potentially lead to modest improvements in staff practices, but these strategies appear to have limited impact on child-level measures [21]. It is also worth noting that the 2019 Canada’s Food Guide was released in January 2019 [41] (about half-way during the 2018–19 data collection period), which could have affected the development of HE policies in childcare centers. One of the changes from the older 2007 Canada Food Guide related to the emphasis of water as the beverage of choice and the elimination of fruit juices as part of the vegetable and fruit group [42]. These changes in dietary guidance could have influenced childcare centers in their development of their policies related to fruit juice and beverages being served. The implementation of HE policy and practice intervention could also be mediated by childcare food provision. Our findings suggest that most children were asked to bring some food (if not all foods) to the childcare center, highlighting the importance of center-level policies targeting foods packed in children’s lunch boxes as well as communicating and supporting parents to address barriers related to HE in the early years. An assessment of childcare food environments in relation to level of implementation (for e.g., proportion of their staff having attended the ATP workshops) could inform whether the ATP scale-up influenced changes in nutrition practices of caregivers or food offered to children among facilities which provide food to children in care.

This study had several strengths including a large sample of childcare providers across urban and rural sites and the inclusion of both manager and staff responses which may present a more reliable depiction of policy and practice changes since the implementation of the AP Standards. Several limitations deserve consideration. First, all policies and PA practices were reported by managers (and in some cases, by staff members as well). Therefore, responses could have been subject to social desirability bias. Yet while the results are expected to be inflated by self-report, it is important to note that this bias was present at both time points and that even with this limitation changes were observed over time. Second, this study was unable to compare the center changes with another province to provide a comparator or control condition for policy comparison. It is therefore possible, as mentioned earlier, that other external contextual factors such as the release of the 24-h Canadian movement guidelines for the early years [12] could have contributed to the observed trends in policies and practices. Finally, this study examined changes over time in policies and practices among group licensed childcare centers in BC, and therefore these results may not be generalizable to family-based childcare centers or preschools with limited hours.

## Conclusions

In summary, the implementation of AP Standards along with capacity-building appeared to facilitate changes in all PA/sedentary policies and some of the HE policies examined among childcare centers in BC. Although PA practice changes were more modest relative to PA policy changes, centers reported more frequent PA supportive practices such as providing children with at least 120 min of daily of AP, 60 min of daily outdoor play and limiting screen time to 30 min following the implementation of the AP Standards and the delivery of ATP across the province. Previous research has linked changes in the childcare environment with children’s nutrition and PA behaviors [43, 44]. Therefore, while the changes in policies reported are encouraging, they also highlight the importance of examining whether such policy experiments lead to objectively measured changes in caregiver practices as well as PA- and HE-related outcomes for children.

## Availability of data and other study materials

The datasets used and/or analysed during the current study available from the corresponding author on reasonable request.

## Abbreviations

AOR: Adjusted Odd’s Ratio
AP: Active Play
ATP: Appetite to Play
BC: British Columbia
CI: Confidence Interval
EPAO-SR: Environment and Policy Evaluation and Observation Self-Report
FMS: Fundamental Movement Skills
HE: Healthy Eating
OR: Odd’s Ratio
PA: Physical Activity
SD: Standard Deviation
